# Caregiver Depression Mediates Caregiver Self Efficacy and Negative Interactions with Care Recipients

**DOI:** 10.1093/geroni/igaf122.4084

**Published:** 2025-12-31

**Authors:** Ayala Tzadikario, Au Myat May, Maya Sakharov, Julian Ortega, Abigail Overstreet, Bárbara Gómez, Hannah Shui-Wah Leung, Rowena Gomez

**Affiliations:** Palo Alto University, Palo Alto, California, United States; Palo Alto Universiety, Palo Alto, California, United States; Palo Alto Universiety, Palo Alto, California, United States; Palo Alto Universiety, Palo Alto, California, United States; Palo Alto Universiety, Palo Alto, California, United States; Palo Alto Universiety, Palo Alto, California, United States; Palo Alto Universiety, Palo Alto, California, United States; Palo Alto Universiety, Palo Alto, California, United States

## Abstract

Previous research has demonstrated that caregiver self-efficacy, the perceived ability to perform according to caregiving demands and make caregiving decisions, may significantly affect caregiver-care recipient relational dynamics (Leung et al., 2020). Additionally, studies have indicated that caregiver depression significantly predicted negative interpersonal outcomes during transitions to facility-based care (Nunez, 2021). The present study examined how caregiver depression may mediate the predictive relationship between caregiver self-efficacy and negative interactions with care recipients in familial caregivers during facility admission transitions for relatives with dementia. As part of a larger randomized controlled trial of the Residential Care Transition Module (RCTM), participants (N = 234; 40 men; 194 women) responded to several questions and measures including: the Caregiver Self-Efficacy Scale, the Center for Epidemiological Studies-Depression Scale, and the Number of Positive and Negative Interactions scale split into six summed categories: Relative Positive, Relative Negative, Staff Positive, Staff Negative, Other Family Members Positive, and Other Family Members Negative. Using Hayes’ PROCESS macro for mediation analysis, the data analyses indicated that caregiver depression fully mediated the relationship between caregiver self-efficacy and negative interactions with care recipients (ps<.05). These results suggest that caregivers with lower self-efficacy may experience increased negative interactions with care recipients because of elevated depression symptoms. These findings may help clinicians better understand the mechanisms contributing to strained caregiver-care recipient relationships during long-term care facility transitions. Furthermore, the findings suggest that interventions targeting depression symptoms may improve interpersonal dynamics between familial caregivers and care recipients during this critical transition period.

